# Further Insights into Metal-DOM Interaction: Consideration of Both Fluorescent and Non-Fluorescent Substances

**DOI:** 10.1371/journal.pone.0112272

**Published:** 2014-11-07

**Authors:** Huacheng Xu, Jicheng Zhong, Guanghui Yu, Jun Wu, Helong Jiang, Liuyan Yang

**Affiliations:** 1 State Key Laboratory of Pollution Control and Resources Reuse, School of the Environment, Xianlin Campus, Nanjing University, Nanjing, China; 2 State Key Laboratory of Lake Science and Environment, Nanjing Institute of Geography and Limnology, Chinese Academy of Sciences, Nanjing, China; 3 College of Resources and Environmental Sciences, Nanjing Agricultural University, Nanjing, China; 4 Key Laboratory of Soil Environment and Pollution Remediation, Institute of Soil Science, Chinese Academy of Sciences, Nanjing, China; Martin-Luther-Universität Halle-Wittenberg, Germany

## Abstract

Information on metal binding with fluorescent substances has been widely studied. By contrast, information on metal binding with non-fluorescent substances remains lacking despite the dominance of these substances in aquatic systems. In this study, the metal binding properties of both fluorescent and non-fluorescent substances were investigated by using metal titration combined with two-dimensional correlation spectroscopy (2D–COS) analysis. The organic matters in the eutrophic algae-rich lake, including natural organic matters (NOM) and algae-induced extracellular polymeric substances (EPS), both contained fluorescent and non-fluorescent substances. The peaks in the one-dimensional spectra strongly overlapped, while 2D–COS can decompose the overlapped peaks and thus enhanced the spectral resolution. Moreover, 2D FTIR COS demonstrated that the binding susceptibility of organic ligands in both NOM and algal EPS matrices followed the order: 3400>1380>1650 cm^−1^, indicative the significant contribution of non-fluorescent ligands in metal binding. The modified Stern-Volmer equation also revealed a substantial metal binding potential for the non-fluorescent substances (log*K_M_*: 3.57∼4.92). As for the effects of organic ligands on metal binding, EPS was characterized with higher binding ability than NOM for both fluorescent and non-fluorescent ligands. Algae-induced EPS and the non-fluorescent substances in eutrophic algae-rich lakes should not be overlooked because of their high metal binding potential.

## Introduction

Dissolved organic matter (DOM) in aquatic environments can interact with metals and consequently affect their toxicity, mobility and bioavailability [1], [2]. Investigation into the binding properties of DOM is necessary to clearly understand the biogeochemical behavior of metals. DOM in waters is generally characterized with heterogeneous organic compositions including fluorescent (i.e., protein-, humic-, and fulvic-like) and non-fluorescent (i.e., carbohydrates, lipids, and lignins) substances [3]–[5]. Thus, a comprehensive study on metal-DOM interaction should include related information on both fluorescent and non-fluorescent substances.

Fluorescence quenching titration is a popular method in the study of metal-ligand interaction [6]–[8]. Saar and Weber [9] compared synchronous fluorescence (SF) quenching titration and ion-selective electrode potentiometry and proposed the former. Ghatak et al. [10] later studied the binding heterogeneity of sediment humic substances and reported more energy binding sites for Fe(III). More recently, through SF quenching titration technique, the heterogeneous metal binding behaviors between leaf litter and soil DOM can be explored [7], [8]. Although widely used, SF technique is not applicable for non-florescent substances, even with these making up a significant proportion of aquatic DOMs [11].

By contrast, Fourier transform infrared spectroscopy (FTIR) can be used to explore non-fluorescent substances [12], [13]. Binding information on non-fluorescnt substances can be obtained by investigating spectral variations in response to metal addition. Thus, the metal binding properties of both fluorescent and non-fluorescent substances can be obtained through the combination of the SF and FTIR techniques.

However, one-dimensional SF and FTIR spectra usually exhibited many overlapped peaks because of the heterogeneous nature of the investigated DOM [6], [10], [14]. Hence, further analysis based solely on the traditional visual inspection approach is laborious and difficult. Recent studies showed that two-dimensional correlation spectroscopy (2D–COS) can be used to solve the peak overlapping problems by extending peaks along the second dimension [12], [14]. This technique can also be used to explore the sequence of metal-sensitive spectral changes through asynchronous map, which benefits the analysis of binding mechanisms [7], [8], [15], [16]. To date, only one study on the application of 2D–COS for exploring the metal binding properties of soil organic matters has been published [16].

Anthropogenically induced eutrophication in recent years has caused the increasing occurrence of cyanobacterial blooms in lakes, especially in eutrophic shallow lakes [17]. Our previous studies have demonstrated that DOM in such algal-rich lakes should consist of natural organic matter (NOM) and algal extracellular polymeric substances (EPS). EPS can be further fractionated into loosely bound EPS (LB–EPS) and tightly bound EPS (TB–EPS) in terms of its binding strength with algal cells [18], [19]. Due to the high reactively, both NOM and algae-induced EPS can bind with metals and consequently play an important role in the toxicity, mobility, and bioavailability of metals.

The present study were therefore aims (1) to investigate the metal-induced spectral variations of both fluorescent and non-fluorescent substances in NOM and algal EPS fractions, and (2) to explore the detailed metal binding behavior and mechanisms by using 2D–COS combined with SF and FTIR techniques. For these purposes, the interactions of a commonly found metal Cu(II) in eutrophic shallow lakes [20] with NOM, LB–EPS and TB–EPS were respectively studied. The results of this study would provide a comprehensive picture of metal-DOM interaction in aquatic environments.

## Materials and Methods

### Ethics statement

No specific permits were required for the described field studies. The location studied is not privately-owned or protected in any way and our studies did not involve any endangered or protected species.

### Sample preparation and EPS extraction

A total of 2.4 L of surface water samples (30 cm from the surface) containing 1.0 g L^−1^ algal biomass were collected from Meiliang Bay, which is located in the northern region of Lake Taihu (Figure S1 in File S1). The samples were collected in the summer-fall period because typical algal blooms occurred during this period [21]. All samples were transported to a laboratory within several hours and firstly filtered through the 0.45 µm polytetrafluoroethylene membranes (Xingya Purification Materials Co., Shanghai, China) for NOM measurement. The residues were carefully scraped and dissolved with a 0.05% NaCl solution to the original volume and centrifuged at 5000 *g* for 15 min, with the liquid collected carefully for measurement of LB–EPS. Afterward, the harvested samples were re-suspended in NaCl solution, heated at 60°C for 30 min and centrifuged at 15,000 *g* for 20 min, with the liquid collected as TB–EPS fraction. More information on sample collection and fractionation procedure can be found elsewhere [18], [19].

### Cu(II) titration and complexation modeling

Titration experiments were carried out by adding 0.1 mol L^−1^ Cu(NO_3_)_2_ solutions to a series of sealed brown vials containing 50 ml of diluted solution [dissolved organic carbon (DOC): 10 mg L^−1^] using an automatic syringe [1], [2], [19], [22]. Metal concentrations ranging from 0 to 100 µmol L^−1^ were obtained in the final solutions by adding no more than 50 µl metal titrant. The pH was maintained at 6.0 through adding 0.1 M HCl or NaOH solution under which no precipitate was formed [1]. All solution samples were shaken for 24 h at room temperature to ensure complexation equilibrium [2]. Afterward, the titrated solutions and freeze-dried samples underwent SF and FTIR analyses, respectively. Each titration experiment was performed in duplicate.

The modified Stern-Volmer equation [23] was applied to estimate the conditional stability constants between metals and SF/FTIR-derived peaks:
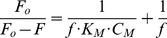
(1)


Here *F* and *F_0_* are the measured SF or FTIR intensities at the metal concentration *C_M_* and the beginning of the titration (i.e., no metal addition), respectively. The parameters *K_M_* and *f* represents the conditional stability constant and the fraction of the initial spectral intensities which correspond to the metal binding.

### Two–dimensional correlation spectroscopy

SF was measured using the Hitachi F–7000 fluorescence spectrometer (Hitachi High Technologies, Tokyo, Japan) in synchronous mode at room temperature by ranging the excitation wavelengths from 200 to 450 nm with a constant offset (60 nm) [7], [24]. The freeze-dried samples were mixed with KBr (sample to KBr ratio of 1∶100, w/w) and then compressed to form a disc. FTIR spectra were obtained by collecting 64 scans at a resolution of 2 cm^−1^ with a Nicolet Nexus 870 FTIR spectrometer [25].

The 2D–COS was produced according to the method of Noda and Ozaki [14]. The metal addition was used as the external perturbation, and thus a set of concentration–dependent SF/FTIR spectra were obtained.

For the perturbation–induced spectral variation y(x, t) as a function of a spectral variable (x) and a perturbation variable (t), the dynamic spectrum 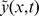 is formally defined as follows:
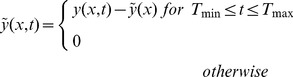
(2)where 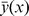 denotes the reference spectra, which is typically the average spectrum and is expressed as 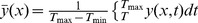. The synchronous correlation spectroscopy can be written as follows:
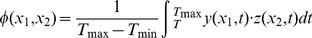
(3)


The asynchronous correlation spectroscopy can be obtained directly from the dynamic spectrum 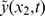
and its orthogonal spectrum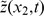.
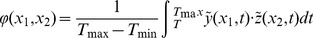
(4)


Prior to 2D–COS analysis, the SF and FTIR spectra were normalized by the summed absorbance from 200 to 450 nm and 400 to 4000 cm^−1^. The noise components were removed by principal component analysis [16], [22]. Then the reconstructed data matrix was progressed using the “2D shige” software (Kwansei Gakuin University, Japan).

## Results and Discussion

### Determination of the organic matters in the eutrophic algae-rich lake

**Figure 1:**
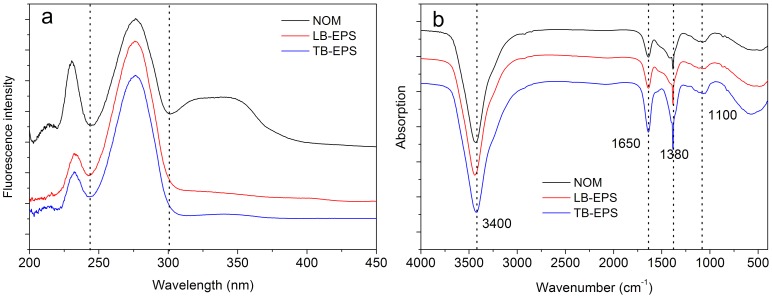
Typical spectral shapes of SF (a) and FTIR (b) for NOM and algal EPS matrix in the eutrophic algae-rich lake.

Figure 1 shows the typical SF and FTIR spectra for the NOM and algal EPS matrices. NOM was characterized with two prominent peaks (230, 280 nm) and one shoulder peak (300∼390 nm), whereas the EPS matrix showed only the prominent peaks (Fig. 1a). Peaks located at 230 and 280 nm were ascribed to tyrosine- and tryptophan-like substances, respectively, whereas peaks ranging from 300 to 390 nm were attributed to humic-like substances [7]. The SF results indicated that the algal EPS mainly contained fluorescent protein-like substances, while not only protein-like but also humic-like substances were observed in NOM. In the previous studies aiming at lab-cultured cynobacterium *M. aeruginosa*, Xu et al. [18] detected the protein-like substances in all EPS fractions but the humic-like substances in only the soluble EPS fractions. And Qu et al. [25] demonstrated that the mainly components in bound and dissolved extracellular organic matters were protein-like substances and protein-like + humic-like substances, respectively. These similar results indicated that the distribution patterns of organic compositions were not form-specific.

As for the FTIR spectroscopy, the NOM and algal EPS matrices were both characterized with four obvious absorption peaks at 3400, 1650, 1380, and 1100 cm^−1^ (Fig.1b). We assigned these bands as follows: the band at 1650 cm^−1^ was ascribed to the CO stretching of amide I in protein compounds, the band at 1380 cm^−1^ to the CH deformations in aliphatic groups, and the bands at 3400 and 1100 cm^−1^ to the stretching vibration of OH and CO in carbohydrate-like substances [16], [26]. The combined results of SF and FTIR showed that DOM in the eutrophic algae-rich lakes, including NOM and algal EPS, definitely contained both fluorescent (230, 280, and 300∼390 nm, and 1650 cm^−1^) and non-fluorescent (3400, 1380, and 1100 cm^−1^) substances.

It should be noted that the organic compositions of EPS matrix were highly dependent on the extraction methods. For example, Liu et al. [27] compared five methods for *M. aeruginosa* EPS extraction and found different fluorescent properties among the five methods. A further study also demonstrated that EPS using cation exchange resin extraction contained more aromatic condensed structures with less acidic functional groups when compared with formaldehyde/NaOH extraction [28]. The heterogeneity in organic compositions exhibits different metal binding ability, which will be discussed in the following section.

### Metal binding properties of the fluorescent substances

#### Changes in one dimensional SF spectra with metal addition

**Figure 2:**
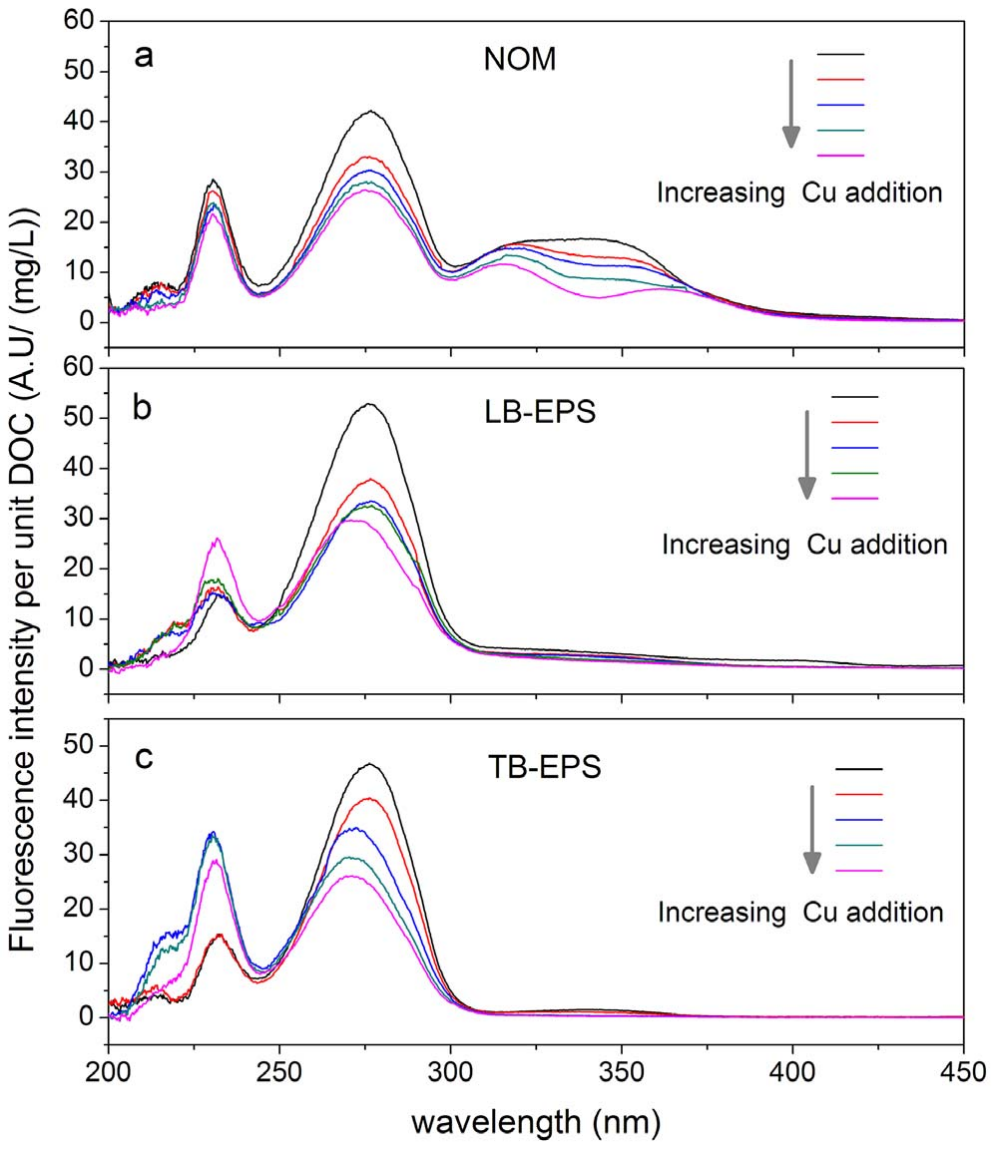
Changes in the one-dimensional SF intensities with Cu(II) addition. The arrows refer to the direction of the increasing metal concentrations.

The effects of Cu(II) addition on the variations of SF spectra are shown in Fig.2. Fluorescence quenching generally occurred with increasing metal addition. This result revealed strong metal binding and the marked modification of the electronic structures of DOM [7], [29]. Further analysis showed that the behavior of spectral changes between NOM and EPS exhibited obvious differences. Monotonic fluorescence quenching was observed for NOM along the entire wavelength region (200∼450 nm), whereas both fluorescence quenching (280 nm) and enhancement (230 nm) were identified in the EPS matrix. The complex formation and the fluorophore-quencher collision were responsible for fluorescence quenching [30], [31], while the fluorescence enhancement was attributed to the changes in quantum yields of tyrosine-like substances in algal EPS matrix caused by high levels of Cu(II) and/or interactions with inorganic and other organic components [2]. Metal-induced fluorescence enhancement was previously reported in other studies but via fluorescence emission and excitation matrix analysis [1], [19], [32].

In addition, the quenching rates and degrees were highly related to the organic ligands. Metal addition caused a sharp and gradual reduction at 250∼300 nm and 300∼370 nm, respectively for NOM. However, the algal EPS matrix exhibited a more pronounced quenching degree than NOM at 250∼300 nm. This result indicated that the protein-like substances in NOM might not be as involved in metal binding as those in the algal EPS matrix [7]. The different quenching rates and degrees observed here indicated that the functional sites for metal binding in the NOM and algal EPS matrices were unevenly distributed.

#### Metal binding sequencing investigated by 2D fluorescence COS

**Figure 3:**
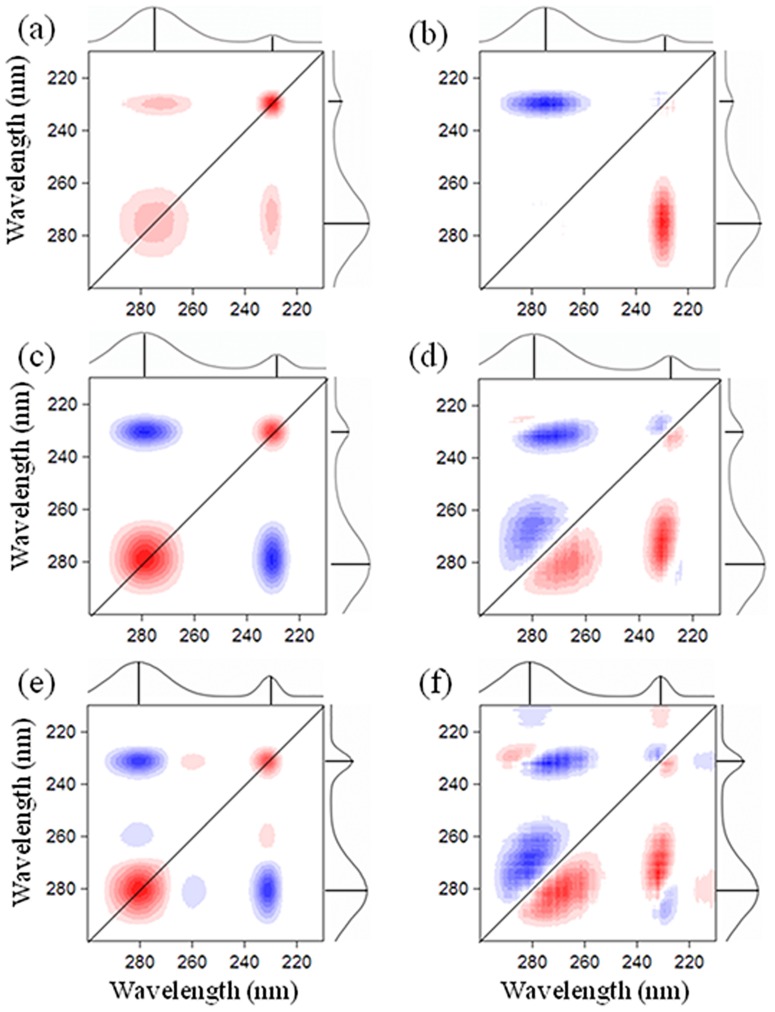
The 2D fluorescence correlation maps generated from 210 to 300 nm region for NOM and EPS matrix with increasing Cu(II) addition. (a) synchronous map for NOM; (b) asynchronous for NOM; (c) synchronous map for LB–EPS; (d) asynchronous for LB–EPS; (e) synchronous map for TB–EPS; (f) asynchronous for TB–EPS. Red represents positive correlations and blue represents negative correlations; higher color intensity indicates a stronger positive or negative correlation.

Although SF can capture heterogeneous quenching degrees and rates, extensive information on binding sites and binding sequence cannot be obtained via the one-dimensional spectral technique. 2D–COS was applied in the present study to explore binding behavior in detail (Fig. 3). The synchronous map displayed two autopeaks at 230/230 and 280/280 nm along the diagonal line for both NOM and algal EPS. The synchronous map is a symmetric spectrum with respect to the diagonal line. The correlation peaks include autopeaks and crosspeaks on the diagonal and off-diagonal lines, respectively. An autopeak represents the overall susceptibility of the corresponding spectral region to the change in spectral intensity as an external perturbation is applied to the system [14], [16], [22]. The intensities of the autopeaks followed the order: 230>280 nm for NOM and 280>230 nm for the EPS matrix. This suggested that tyrosine-like substances in NOM were more susceptible to metal addition compared with the tryptophan-like substances, whereas the tryptophan-like substances in algal EPS matrix exhibited higher metal sensitivity than the tyrosine-like substances [8], [14].

A crosspeak in synchronous map represents the simultaneous or coincidental changes of two different spectral variables. NOM and algal EPS were respectively characterized with positive and negative crosspeak at 280/230 nm. This characterization implies that tyrosine- and tryptophan-like substances in NOM covary in their interaction with metal addition, whereas those in algal EPS might vary with opposite directions. Consistent with our previous observations (Fig. 2), NOM demonstrated monotonic quenching effects, whereas the algal EPS matrix demonstrated both fluorescence quenching and enhancement.

An asynchronous map reveals the sequential or successive changes in spectral intensities with increasing metal addition. As shown, one and three negative crosspeaks were observed above the diagonal line for the NOM and algal EPS matrices, respectively. Based on Noda's rule [14], the sequences of binding affinities followed the order: 230>275 nm for NOM, 265>280>225>230 nm for LB–EPS and 270>280>230>232 nm for TB–EPS. The asynchronous maps revealed that metal binding in NOM took place at the shorter wavelengths (∼230 nm) prior to that at the longer wavelength range (>265 nm); while for the algal EPS matrix, meals binding first occurred at the longer wavelengths. As indicated by these results, the preferential metal ligands in NOM and algal EPS were tyrosine- and tryptophan-like substances, respectively. In addition, the asynchronous maps also showed that the wavelength 230 nm in the one-dimensional SF spectra overlapped with 230 and 232 nm, and the wavelength 280 nm overlapped with 270, 275 and 280 nm. Unlike the one-dimensional spectral technique, 2D–COS can enhance spectral resolution and yield extensive detailed binding information.

### Information on metal binding with both fluorescent and non-fluorescent substances

FTIR can be used to characterize non-fluorescent substances [12], [13]. The absorption variations of the normalized one-dimensional FTIR spectra of NOM and algal EPS in response to Cu(II) addition are given in Figure S2 in File S1. Although four absorption peaks were obviously observed at 3400, 1650, 1380 and 1100 cm^−1^, the one-dimensional FTIR was strongly overlapped and information on the interrelationship among metal-sensitive functional groups cannot be obtained.

**Figure 4:**
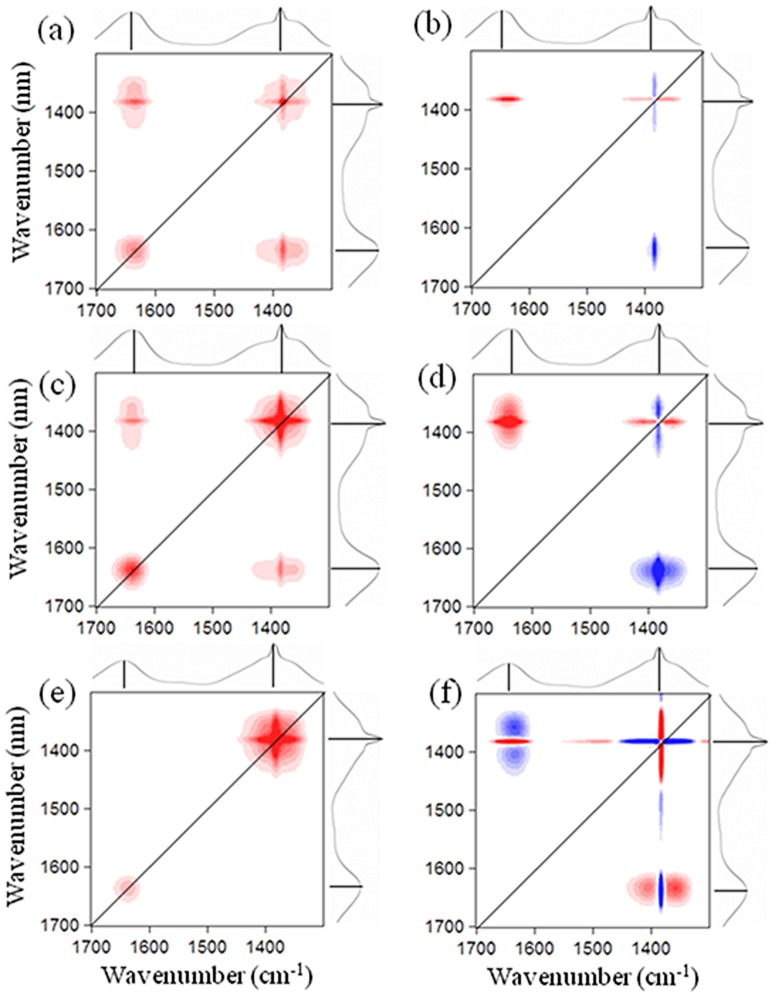
The 2D infrared correlation maps generated from 1300 to 1700 cm^−1^ region for NOM and EPS matrix with increasing Cu(II) addition. (a) synchronous map for NOM; (b) asynchronous for NOM; (c) synchronous map for LB–EPS; (d) asynchronous for LB–EPS; (e) synchronous map for TB–EPS; (f) asynchronous for TB–EPS. Red represents positive correlations and blue represents negative correlations; higher color intensity indicates a stronger positive or negative correlation.

Therefore, 2D–COS was applied to explore in detail the binding information on fluorescent and non-fluorescent substances (Fig.4). We mainly focused on the region between 1700∼1300 cm^−1^ because this spectral region usually contains the major bands of the amide, carboxylic, ester, and carbohydrate functional groups [12], [16]. Both NOM and algal EPS matrices were characterized with two autopeaks located at 1380/1380 and 1650/1650 cm^−1^, with the former exhibiting higher intensity than the latter. Combined with the synchronous maps of the 4000–400 cm^−1^ region (Figure S3 in File S1), the peak at 3400 cm^−1^ was more susceptible to Cu(II) addition, followed by the peak at 1380 cm^−1^ and 1650 cm^−1^. This result revealed the substantial contributions of non-fluorescent substances (3400, 1380 cm^−1^) in metal binding. Zhang et al. [31] also emphasized the metal binding potential of non-fluorescent carboxyl and hydroxyl functional groups in biofilm EPS, although no further evidence was given in their studies.

By contrast, the asynchronous maps revealed that the sequencing of binding affinities of the peaks followed the order: amide I in proteins (1650 cm^−1^)> lignin and aliphatic CH (1380 cm^−1^) for NOM and LB–EPS, and amide I in proteins (1650 cm^−1^)> lignin and aliphatic CH (1380 cm^−1^)> cellulose CH_2_ (1420 cm^−1^) for TB–EPS [14]. Therefore, metal Cu(II) preferentially bound with the fluorescent protein-like substances, followed by the non-fluorescent liquid- and carbohydrate-like substances, regardless of the difference in DOM fractions (i.e., NOM, LB–EPS and TB–EPS).

### Metal binding abilities of both fluorescent and non-fluorescent substances

The conditional stability constants (log*K_M_*) based on the modified Stern-Volmer equation are tabulated in Table 1. The log*K_M_* values for EPS matrix were calculated within the range from 4.66 to 5.03, which were slightly higher than those reported in Lee et al. [28] but lower than those in McIntyre and Gueguen [33]. The heterogeneous binding capacities were partly attributed to the different organic compositions and functional groups, which were highly dependent on the extraction methods [28]. Further analysis showed that the stability constants between NOM and EPS exhibited obvious differences. Specifically, the log*K_M_* of tryptophan-like substances in NOM (4.51±0.06) was lower than that in the LB–EPS and TB–EPS fractions (>4.66±0.05). The results indicated that the tryptophan-like substances in the algal EPS exhibited higher binding abilities than those in NOM [19].

**Table 1:** Conditional stability constants (log*K_M_*) for SF/FTIR-derived peaks binding to Cu(II) as calculated using the modified Stern-Volmer equation.

Samples	Wavelength (nm)	log*K_M_*	*R* ^2^	Samples	Wavenumber (cm^−1^)	log*K_M_*	*R* ^2^
	230	4.69±0.04	>0.87		1100	3.57±0.04	>0.93
NOM	275	4.51±0.06	>0.72	NOM	1380	4.27±0.05	>0.88
					1650	4.62±0.08	>0.71
	230	Not modeled			1100	4.35±0.03	>0.92
LB–EPS	265	4.94±0.07	>0.51	LB–EPS	1380	4.59±0.08	>0.71
	280	4.66±0.05	>0.84		1650	4.79±0.04	>0.86
	230	Not modeled			1100	4.74±0.05	>0.80
	233	Not modeled			1380	4.92±0.03	>0.86
TB–EPS	270	5.03±0.04	>0.88	TB–EPS	1420	4.26±0.10	>0.62
	280	4.74±0.03	>0.95		1650	4.96±0.07	>0.76

For the FTIR-derived peaks, the peak at 1650 cm^−1^ showed a higher log*K_M_* value than the peaks at 1100, 1380 and 1420 cm^−1^ for NOM (4.62±0.08 vs. <4.27±0.05), LB–EPS (4.79±0.04 vs. <4.59±0.08) and TB–EPS (4.96±0.07 vs. <4.92±0.03). This result implies that the binding ability of fluorescent proteins is stronger than that of the non-fluorescent liquids and carbohydrates. The calculated log*K_M_* values were consistent with the results of asynchronous correlation spectroscopy, thus providing evidence for the feasibility of 2D–COS in the investigation of metal-DOM interaction. Further analysis showed that the algal EPS matrix exhibited a higher log*K_M_* value than NOM for both the fluorescence proteins (>4.79±0.04 vs. 4.62±0.08) and the non-fluorescence liquids and carbohydrates (>4.26±0.10 vs. <3.57∼4.27). Therefore, the algal EPS matrix is expected to play a more important role than NOM in the migration and detoxification of heavy metals in eutrophic algae-rich lakes.

### Significance of this study

Both fluorescent and non-fluorescent substances were detected in the DOM found in lakes (Fig. 1). Previous studies on metal-DOM interaction mainly focused on fluorescent substances [1], [34], [35], lacking the related information on non-fluorescent substances. Non-fluorescent substances may sometimes even occupy a significant proportion in aquatic environments [11]. The contribution of non-fluorescent substances in metal binding was investigated for the first time in this study, which is an important complement to the research scope of metal-DOM interaction.

Algal blooms have become common in recent years because of climatic changes and nutrient enrichment [17]. This phenomenon has made the algae-induced EPS an important contributor to the DOM pool [33]. Although studies on metal binding with NOM have been carried out [2], [8], information on the metal-EPS interactions in eutrophic algae-rich lakes remains insufficient. This information would help provide a comprehensive picture of metal-DOM interactions in aquatic environments. The higher metal-EPS binding capacities observed in this work highlighted the ecological importance of algal EPS in the toxicity, mobility, and bioavailability of metals because the toxicity of metals is related to free iron activity [36].

Although the metal binding degrees and rates can be explored through a one-dimensional spectral analysis, the interrelationships among different organic ligands cannot be obtained. The 2D–COS applied in this study clearly demonstrated the heterogeneous metal binding sites between NOM and algal EPS. More importantly, 2D–COS can capture the binding sequencing of metal-sensitive spectral variations, which could improve our understanding about metal binding mechanisms. This also allows the scope of 2D–COS to be unlimited in the fields of metal binding within lake ecosystems [7]. The interaction of DOM with other objects (i.e., nanoparticles and persistent organic pollutants) in selected aquatic environments (i.e., groundwater and wastewater) can also be explored by 2D–COS in future studies. External perturbation need not be limited to metal concentration but may include other parameters, such as temperature, pressure, and pH.

## Conclusions

DOM in eutrophic algae-rich lakes was fractionated into NOM, LB–EPS, and TB–EPS fractions. Both fluorescent and non-fluorescent substances were determined in these fractions. Although fluorescence titration was an effective method for investigating metal-ligand interaction, FTIR was able to determine both fluorescent and non-fluorescent substances. 2D–COS can enhance spectral resolution and thus can be used to explore detailed binding properties such as binding sites and binding sequencing. The metal binding properties of non-fluorescent substances were quantified, and the significance of the algal EPS matrix in metal detoxification was emphasized. In sum, 2D–COS combined with SF and FTIR analysis is an effective approach to explore the metal binding properties of both fluorescent and non-fluorescent substances.

## Supporting Information

File S1 **Supporting figures.** Figure S1. The map of Lake Taihu with the location of the sampling station; Figure S2 The variations of the normalized one-dimensional FTIR with metal addition; Figure S3 The 2D synchronous correlation maps generated from 4000–400 cm^−1^ region of the FTIR spectra for NOM and algal EPS.
(DOC)
