# Supplementary material for: Further Insights into Metal-DOM Interaction: Consideration of Both Fluorescent and Non-Fluorescent Substances
Source: PLoS One. 2014 Nov 7;9(11):e112272. doi: 10.1371/journal.pone.0112272 (PMC4224434; doi:10.1371/journal.pone.0112272)
Supplement: File S1 — Supporting figures. Figure S1. The map of Lake Taihu with the location of the sampling station; Figure S2 The variations of the normalized one-dimensional FTIR with metal addition; Figure S3 The 2D synchronous correlation maps generated from 4000–400 cm−1 region of the FTIR spectra for NOM and algal EPS. (DOC) [file pone.0112272.s001.doc]

**Supporting Information**

**Further** **insights int****o** **metal-DOM interaction:** **Consideration of both f****luorescent and non-fluorescent substances**

Huacheng Xu^1,2*^, Jicheng Zhong^2^, Guanghui Yu^2,3^, Jun Wu^4^, Helong Jiang^2^, Liuyan Yang^1*^

1. State Key Laboratory of Pollution Control and Resources Reuse, School of the Environment, Xianlin Campus, Nanjing University, Nanjing 210023, China.

2. State Key Laboratory of Lake Science and Environment, Nanjing Institute of Geography and Limnology, Chinese Academy of Sciences, Nanjing 210008, China.

3. College of Resources and Environmental Sciences, Nanjing Agricultural University, Nanjing 210095, China.

4. Key Laboratory of Soil Environment and Pollution Remediation, Institute of Soil Science, Chinese Academy of Sciences, Nanjing, 210008, China

^*^Corresponding author. E-mail: [hcxu@niglas.ac.cn](mailto:hcxu@niglas.ac.cn) (H.C. Xu); [yangly@nju.edu.cn](mailto:yangly@nju.edu.cn) (L.Y. Yang).


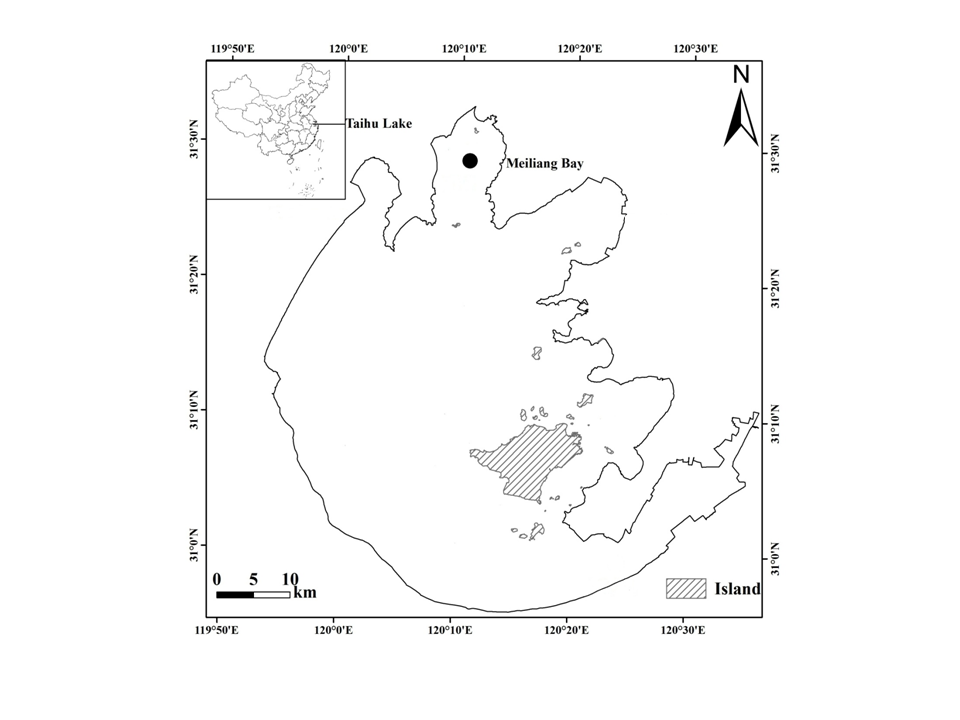


Figure S1. Map of Lake Taihu with the location of the sampling station


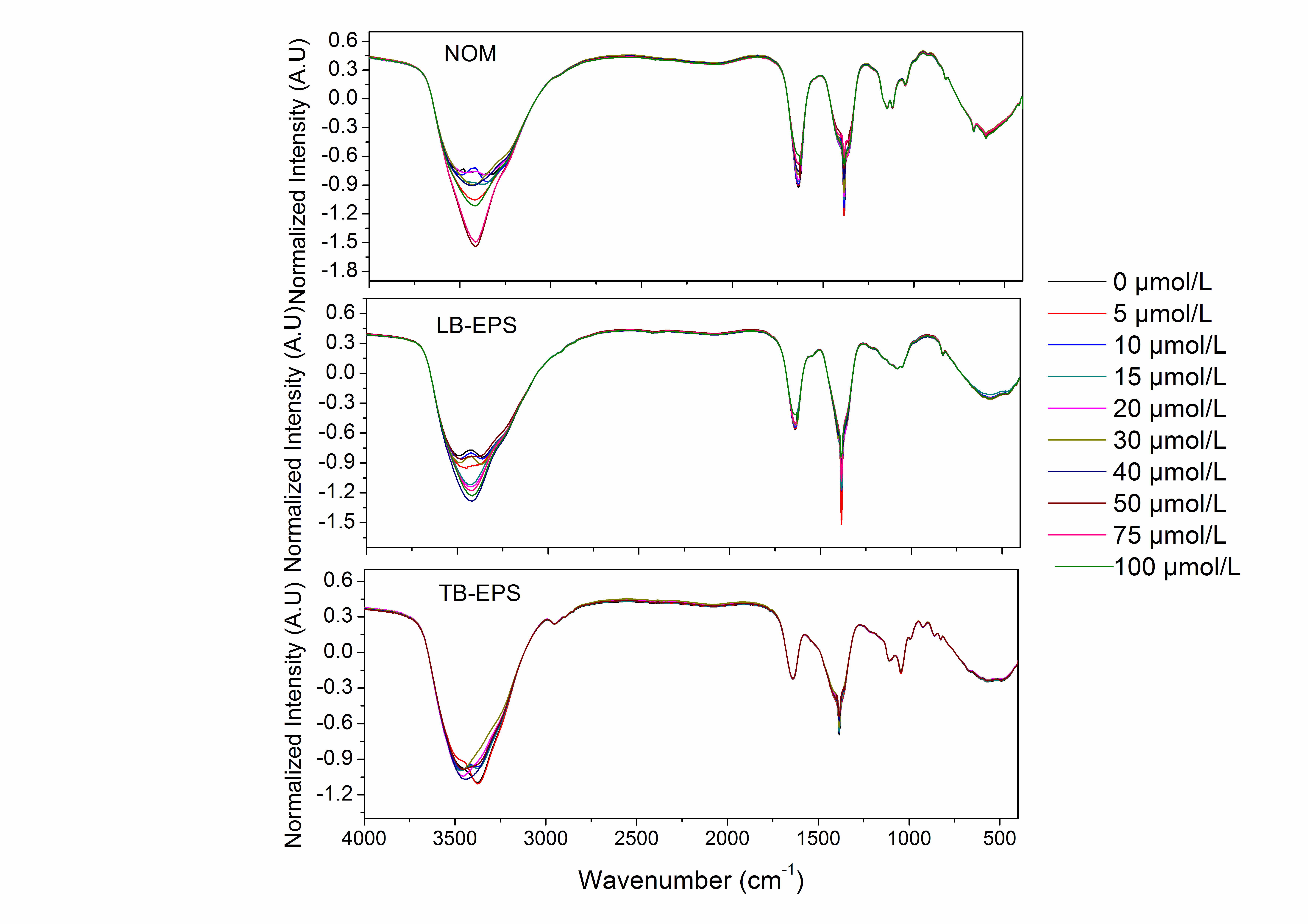


Figure S2. Changes in the normalized one-dimensional FTIR spectra with Cu addition


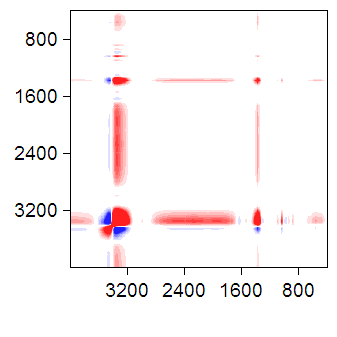


NOM


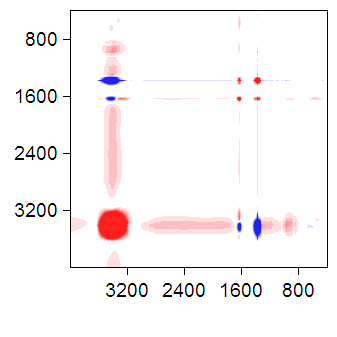


LB-EPS


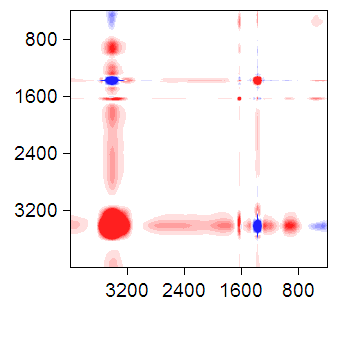


TB-EPS

Figure S3. Synchronous 2D correlation maps generated from 4000–400 cm^-1^ region of the FTIR spectra for NOM and algal EPS.
